# Covid-19, Lockdown and Self-Isolation: Evaluation of Deliberate Self-Harm Admissions

**DOI:** 10.3389/fpsyt.2021.662885

**Published:** 2021-05-17

**Authors:** Callum Shields, Jack Bernard, Omer Idris Mirza, David Reeves, Adrian Wells, Anthony Heagerty

**Affiliations:** ^1^Manchester University Foundation Trust, Manchester, United Kingdom; ^2^Division of Population Health, Health Services Research and Primary Care, Manchester, United Kingdom; ^3^School of Psychological Science, Section of Clinical and Health Psychology University of Manchester Rawnsley Building, Manchester, United Kingdom; ^4^Division of Cardiovascular Sciences, Faculty of Biology, Medicine and Health, University of Manchester Core Technology Facility, Manchester, United Kingdom

**Keywords:** COVID-19, self harm, lockdown, mental health, stress, deliberate self harm, suicide

## Abstract

**Background:** COVID 19 is still presenting a clear and dynamic global threat. The United Kingdom remains one of the hardest hit countries from the pandemic. In January 2021 parliament announced that the UK will be entering a full national lockdown. This paper explores what effect lockdown measures had on rates of deliberate self-harm presentations to one NHS trust in Manchester UK.

**Methods:** This paper compared the number of cases of deliberate self-harm which presented to the emergency department of Manchester Royal Infirmary for March-May in 2018, 2019 and 2020. This was achieved by utilising coding from emergency department data and reviewing hospital records surrounding each case.

**Results:** 2018 recorded a total of 101 admissions as a result of DSH with all causes admissions of 8,514 making the proportions of admissions due to self-harm 1.19%. In 2019, 9,038 patients were admitted, of these, 130 (1.44%) were identified as DSH. In 2020 the total number of admissions fell to 5,676 with 118 admitted due to self-harm, representing 2.08% of admissions. The absolute number of admissions remained stable however the proportion of admissions due to self-harm was significantly higher in 2020 (*p* < 0.001). Other significant findings include a higher proportion of male admissions compared to females in 2020 (58.5%) and a decrease in the normal of cases relating to paracetamol overdose in 2020.

**Discussion:** The findings demonstrated by this study do not indicate that lockdown is an absolute risk for DSH behaviours however it does illustrate the stable nature of these cases despite and dramatic decline in all cause admissions. The rate of increase of deliberate self-harm accelerated significantly between March and May in 2020. Steps must be taken to avoid a similar situation following the 2021 lockdown and beyond – focus on improving access to certain virtual services may help to achieve this goal.

## Background

COVID 19, the disease caused by the novel variant of the Sars-Cov-2 virus is still presenting a clear and dynamic global threat. Despite the glimmers of hope offered by the roll out of several vaccines, the virus is still sweeping through many international communities (1).

During the first wave of the pandemic in spring 2020 the UK Government enforced the first nation-wide lockdown to help combat the spread of the virus. This led to severe social restrictions, prohibiting mixing between households and a blanket closure of almost all hospitality and leisure industries. These measures remained in force from March-May after which gradual relaxation of the rules occurred (2). Moving forward to 2021, the United Kingdom (UK) remains one of the hardest hit countries from the pandemic and figures show a peak incidence of over 60,000 daily cases in January. Additionally, a near 25,000 hospital admissions due to the virus occurred in the first week of 2021, when coupled with the usual winter pressures exerted on the NHS, there was growing concern that our healthcare system would exceed breaking point (3). It is for this reason Parliament announced on the 4th of January that the UK will be entering a full national lockdown akin to the measures enforced during the first peak of the pandemic in spring 2020 (4). These restrictions are to be in place until at least April and although a provisional date of June 21st has been established for a return to normal, future lockdown periods remain a real possibility (5). This is evidenced by the emergence of multiple variant strains of COVID-19, the possibility of vaccine resistance and the need to accommodate normal winter pressures (6). With the prospect of further periods of strict health protection laws on the horizon, it is prudent to reflect on the original 2020 lockdown to examine what effects it had on the mental health of the population. Indeed, many papers have examined the impact these restrictions had on mental health of the population generally (7–11). Less have commented on how this impact has translated into severe manifestations such as suicidal behaviours and deliberate self-harm (DSH).

Historically, it has been documented that extreme social phenomena such as pandemics increase the burden on mental health. During the Spanish flu pandemic, the literature reports that one repercussion stemming from this is higher than normal levels of suicidal behaviours (12). Many papers have attempted to postulate the underlying aetiology behind these spikes in morbidity. Accounts from the time comment on the culpability of an acute influenza induced delirium or psychosis (13). Whereas, more contemporary papers state that societal factors such as loss employment or curbs on social freedoms are more likely responsible (12, 14). Furthermore, a recent study published in the Lancet highlighted the potential effects of a long-COVID syndrome on psychiatric disorders at 6 months post infection. This paper reported a statistically significant hazards ratio of 1.47 in the development of mood disorders amongst COVID patients compared to those with seasonal influenza (15). Given these links, this paper examines the effects of the initial lockdown period in the UK and the number of DSH admissions. This was done with reference to the World Health Organisation definition of self-harm:

“an act with non-fatal outcome, in which an individual deliberately initiates a non-habitual behaviour that, without intervention from others, will cause self-harm, or deliberately ingests a substance in excess of the prescribed or generally recognised therapeutic dosage, and which is aimed at realising changes which the subject desired via the actual or expected physical consequences” (16).

### Primary Aim

To determine what, if any, effect lockdown measures had on the number of deliberate self-harm admissions.

### Methods

We compared the number of admissions from the Emergency Department of Manchester University Foundation Trust (Manchester Royal Infirmary) from March 1st to May 31st 2018, 2019, and 2020 and identified patients with a diagnosis of self-harm. This study period was chosen to reflect the most stringent lockdown restrictions present in the UK, specifically referencing the prohibition of social mixing between households hence representing the greatest degree of isolation (2).

Many papers have utilised survey methods to establish a general deteriorative trend in mental health during the pandemic (7–11). This study therefore focused specifically on cases which required admission to hospital for further treatment. This criterion was chosen to allow the data to embody severe cases of DSH over the study period. This was to allow reflection on the metric of self-harm behaviours of a degree severe enough to warrant admission.

We used local emergency departmental coding data to identify all cases coded as DSH for the study periods, as well as all cause presentations for the same period.

Inclusion criteria for the study were as follows:

1. Age over 16, this study focused solely on attendances to the adult emergency department.
2. Attendance coded as “Overdose and poisoning,” “Self-harm” or “Major trauma” on the emergency department admission sheet.
3. Attendances that were of a degree/severity to warrant admission into the hospital.
4. Reference to deliberate intent of self-harm contained within the emergency department admission summary sheet. This was achieved by examining the “nurse triage” or “clinician's comments” section of the summary sheet and identifying which patients had acted with the intent to cause harm to themselves. This filtered out presentations for accidental injuries or overdoses, for example when an individual had mistakenly taken too many paracetamol tablets.

From this dataset we then used hospital EPR systems to extract key facets of each presentation – length of stay, mode of self-harm, intensive care involvement, death – to compare each year. The mode of self-harm contained several categories defined below:

1. Major trauma – Involved serious injuries from self-inflicted traumas most commonly jumping from heights or stabbings.
2. Self-mutilation – Injuries of a lesser severity than major trauma such as superficial incisions or wounds.
3. Household products – Involving ingestion of items found within the house such a bleach.
4. Alcohol – Cases which involved alcohol.
5. Recreational drugs – Cases which involved the use of drugs such as cannabis/cocaine.
6. Medication – Cases involving prescription medication such as anti-depressants, analgesia or any other pharmaceutical drug. The cases which referenced paracetamol were also included within this category.
7. Paracetamol – Cases specifically referencing the use of paracetamol.

Each case reported in the results represented a unique admission. Some cases did contain more than one mechanism of injury e.g., self-inflicted wound and paracetamol overdose. This provides explanation for the mismatch between the total number of cases and the overall counts for the underlying mechanism.

### Statistical Analysis

Analysis focused principally on comparing numbers and rates of DSH admissions between years (2018, 2019, and 2020) and calendar months (March, April, May), and in relationship to patient characteristics. The admissions data was in the form of counts and with the exception of age the factors of interest were categorical, for which statistical inference was undertaken using Pearson Chi^2^ analysis; to test for age differences we used one-way analysis of variance.

To examine if the characteristics of admitted patients differed between years, we pooled the data across the 3 months of observations within each year prior to analysis. When testing for differences in admission numbers and rates between years, to minimise multiple testing we first conducted an overall test for equality across years within months and only if that was rejected went on to test each month separately. An alpha value for statistical significance of 5% was used throughout.

## Results

### Patient Demographics

The total number of admissions for DSH across the 3 years were 101, 130 and 118 for 2018, 2019, and 2020, respectively. These cases were then stratified for age, sex, ethnicity and marital status to discern any meaningful demographic differences across the study periods. The results are summarised in Table 1.

**Table 1:** Demographic differences across study years by age, sex, marital status and ethnic category for DSH admission cohort.

**Year**	**2018**	**2019**	**2020**	**Mean**
Age (average)	34.4	36.0	39.4	36.6
**Sex** ***N*****(%)**				
Male	39 (38.6)	56 (43.1)	69 (58.5)	54.7 (46.7)
Female	62 (61.4)	74 (56.9)	49 (41.5)	61.7 (53.3)
**Marital status** ***N*****(%)**				
Single	82 (81.2)	93 (71.5)	92 (78.0)	89 (76.9)
Married	6 (5.9)	11 (8.5)	10 (8.5)	9 (7.6)
Divorced	2 (2.0)	6 (4.6)	2 (1.7)	3.3 (2.8)
Widowed	2 (2.0)	3 (2.3)	0 (0.0)	1.7 (1.4)
Not stated	9 (8.9)	17 (13.1)	14 (11.8)	13.3 (11.3)
**Ethnic category** ***N*****(%)**				
British	77 (76.2)	88 (67.7)	90 (76.3)	85 (73.4)
Irish	1 (1.0)	2 (1.5)	0 (0.0)	1 (0.8)
African	3 (3.0)	2 (1.5)	1 (0.8)	2 (1.8)
Caribbean	2 (2.0)	1 (0.8)	1 (0.8)	1.3 (1.2)
Chinese	0 (0.0)	1 (0.8)	3 (2.5)	1.3(1.1)
Indian	0 (0.0)	2 (1.5)	0 (0.0)	0.7 (0.5)
Pakistani	2 (2.0)	7 (5.4)	4 (3.4)	4.3 (3.6)
Mixed ethnicity	0 (0.0)	4 (3.1)	1 (0.8)	1.7 (1.3)
Any other ethnic background	8 (7.9)	13 (10.0)	10 (8.5)	10.3 (8.8)
Not stated	8 (7.9)	10 (7.7)	8 (6.8)	8.7 (7.5)

The groups were well-matched in terms of marital status and ethnic category with even distribution across the 3 years. A Pearson Chi^2^ test comparing the percentage single (vs. any other marital status) between the 3 years was not statistically significant (Chi^2^ 3.16, df = 2, *p* = 0.206), nor was a comparison of the percentage of British or Irish ethnicity, vs. any other (Chi^2^ 2.39, df = 2, *p* = 0.302). However, mean age differed between the years (F = 3.65, df = 2:346, *p* = 0.027) with the 2020 group being somewhat older, as did the ratio of male to female admissions (Chi^2^ 9.89, df = 2, *p* = 0.007), with an increase in male representation in 2020.

### DSH Admissions March 1st – May 31st 2018

**Figure 1:**
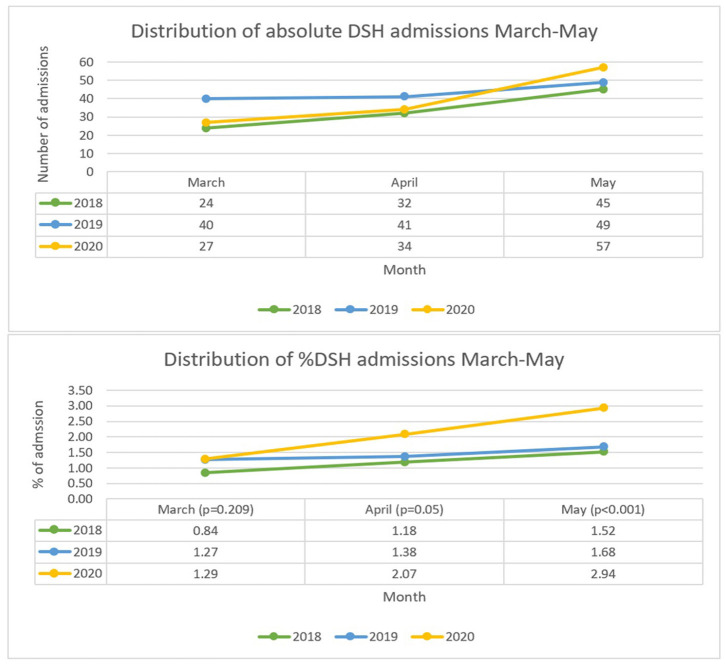
Graphs comparing absolute and proportion of DSH by month and year.

**Figure 2:**
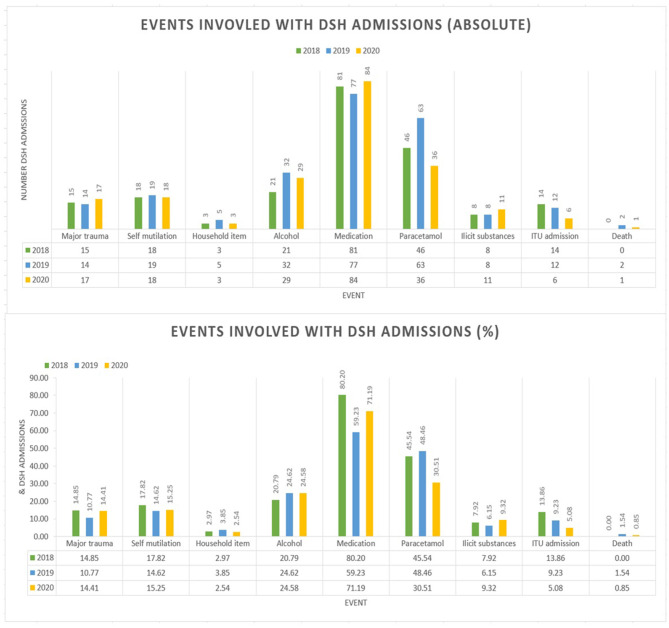
Graphs showing events involved with DSH admissions by year.

A total of 101 admissions as a result of DSH with all causes admissions of 8,514. This makes the proportions of admissions due to self-harm 1.19% with a monthly distribution of 0.84% (March), 1.18% (April) and 1.52% (May) – see Figure 1 for full comparison. The average age in this group was 34.4 years and the average length of admission was 2.6 days. There were no deaths within this cohort however 14 (13.9%) of cases resulted in ITU/HDU input. In terms of coding, 15 (14.9%) were classified as major trauma, 18 (17.8%) as self-mutilation, 3 (3.0%) involved household items, 21 (20.8%) referenced alcohol involvement whilst 8 (7.9%) involved recreational substances. Medication was involved in 81 (80.2%) cases with 46 (45.5%) of these relating to paracetamol overdoses – see Figure 2 for comparison of mechanisms of harm by year.

### DSH Admissions March 1st – May 31^st^ 2019

In 2019, a total of 9,038 patients were admitted via the emergency department, 130 were identified as being due to self-harm thus equating to 1.44% of admissions. Broken down by month to 1.27% (March), 1.38% (April) and 1.68% (May) – Figure 1. In this cohort the average age was 36 years, and the average length of hospital admission was 5.4 days. There were 2 (1.5%) deaths within this group and 12 (9.2%) had ITU/HDU involvement. Major trauma was cited in 14 (10.8%) of cases, 19 (14.6%) as self-mutilation, 5 (3.9%) involved household products. Alcohol was implicated in 32 (24.6%) of cases and recreational drugs in 8 (6.2%). A total of 77 (59.2%) cases involved medication overdoses with 63 (48.5%) involving paracetamol – Figure 2.

### DSH Admissions March 1st – May 31st 2020

During lockdown in 2020 the total number of admissions fell to 5,676 a 33% decrease from 2018 and 37% decrease from 2019. The number diagnosed with self-harm was 118 representing 2.08% of all cause admissions – 1.29% (March), 2.07 (April), 2.94 (May) – Figure 1. The average age was 39.5 years and length of stay was 3.3 days. One (0.9%) death resulted from the self-harm in this year and 6 (5.1%) required escalation to HDU/ITU care. During 2020, major trauma accounted for 17 (14.4%) cases, 18 (15.3%) from self-mutilation and 3 (2.5%) from household items. Alcohol was involved in 29 (24.6%) of cases and 11 (9.3%) featured recreational drugs. Medication overdose was cited in 84 (71.2%) cases with 36 (30.5%) related to paracetamol overdoses – Figure 2.

### Comparison of Years

#### Absolute Numbers of DSH Admissions

Numbers of DSH admissions in the months of March, April and May did not differ significantly between years (overall Chi^2^ 7.51, df = 6, *p* = 0.277). By contrast, total admissions for all causes differed between years, both overall and within each month separately (overall Chi^2^ 882.1, df = 6, *p* < 0.001; March Chi^2^ 219.4, df = 2, *p* < 0.001; April Chi^2^ 408.8, df = 2, *p* < 0.001; May Chi^2^ 254.0, df = 2, *p* < 0.001). The main contributing factors were the large reductions in all-cause admissions in April and May 2020 compared to 2019.

#### Proportions of DSH Admissions

Analysed as proportions of all emergency department admissions, rates of admissions for DSH differed between years (overall Chi^2^ 22.9, df = 6, *p* < 0.001). Under month-specific analysis, rates for March did not differ between years (Chi^2^ 3.14, df = 2, *p* = 0.209); but did differ for April (Chi^2^ 5.86, df = 2, *p* = 0.05), and May (Chi^2^ 13.9, df = 2, *p* < 0.001), largely driven by increased rates in April and May 2020 of 50 and 75%, respectively, compared to 2019.

#### Events Involved in DSH Admissions

Comparison of the rates at which each type of event was involved in DSH admissions found a significant difference between years for medication (Chi^2^ 12.04, df = 2, *p* = 0.002) and paracetamol (Chi^2^ 9.14, df = 2, *p* = 0.01) related event. It is notable that compared to 2019, the rate of medication overdose increased while paracetamol overdose decreased, despite the latter representing a major component of the former.

## Discussion

The number of admissions for DSH remained relatively steady across the study years. This indicates that, for the study cohort, the absolute risk of DSH did not change considerably during the lockdown period compared with previous years. One interesting facet to this finding however is the steadfastness of DSH admissions despite a radical decline in all cause admissions. As mentioned above, 2020 saw a respective drop of 33 and 37% in all cause admissions compared to 2018 and 2019. This has led to a statistically significant increase in the proportion of admissions due to DSH in 2020 compared to previous years. The large fall in overall medical admissions was a feature observed by many hospitals during the lockdown period and from a patient perspective may be attributed to fear of catching Covid-19 and a desire by many not to “bother” the NHS during a time of crisis (17). Furthermore, hospitals may have employed more stringent admission criteria than usual due to the potential bed crisis that loomed shortly after the first lockdown began. Nonetheless, even with this significant reduction in all cause admissions, the number of DSH admissions proved unmalleable. This resistance may go some way in exemplifying the underlying aetiology of DSH behaviours. One of the driving factors behind an individual's desire to self-harm emanates from a help seeking mechanism. Indeed, a review by Edmondson et al. discerned that 87% of questionnaire studies linked self-harm practice as a way of expressing emotional pain to others (18). So, acknowledgement of the harm by a third party is an integral part of the underlying disease process for some individuals and hence explains the continued impulse to attend the emergency department. One of the main factors allaying people's desire to attend hospital during the pandemic is due to anxiety around catching Coronavirus. It is well-documented that individuals displaying suicidal behaviours, such as DSH, are more likely to participate in self-destructive patterns and have a lower regard for self-worth (19, 20). This unrelenting internal view directly opposes the COVID catching anxiety experienced by other patients, thereby helping them to overcome this barrier to attendance. Both reasons help to explain why the denominator of all causes admissions has reduced whilst the numerator for DSH admissions has remained stable.

A similar study conducted in Birmingham UK demonstrated an absolute increase in the presentations of deliberate self-harm to the emergency department. This study noted a rise in the percentage of these presentations from 1.98% to 3.69% (*p* < 0.001) when comparing 2019 to 2020 (21). These results may suggest a substantive negative impact of lockdown on mental health. However, this percentage increase only translates to an extra 10 cases overall which, as the authors state, could be attributed to normal year on year variation rather than directly because of lockdown. Additionally, data from Oxford and Derby conversely showed a 37% decline in the mean weekly number of self-harm presentations in 2020 compared to 2019. This equated to an average reduction of 18 cases per week during the lockdown period (22). Given that during this period the UK remained in a nationally standardised lockdown protocol it is unlikely that either local restrictions or fear of COVID itself account for these geographical differences. A possible explanation for this discord could be related to the regional variance of social factors such as economic instability. Financial security is a commonly cited variable linked with mental health, especially during a pandemic era (23). A report from the Office of National Statistics (ONS) highlighted the disparity of furloughed employees by geographical region. With Birmingham totalling 416,900, followed by 407,900 in Manchester, 103,000 in Oxford 37,500 for Derby (24). In addition to furlough there is also large regional variation in unemployment during the first half of 2020, again with the West Midlands (Birmingham) most severely affected at a rate of 4.6%. This compares to 3.7% in the North West (Manchester), 3.3% in the South West (Oxford) and 4.5% in the East Midlands (Derby) (25). This variation in the levels of financial uncertainty experienced by individuals due to lockdown may go some way in explaining the difference in mental resilience noted between locations within the UK.

On an international level a study from Japan which also centred around suicidal behaviours found that during the final quarter of 2020, suicide rates increased significantly compared with the same quarter in the previous 4 years. The maximal effect was demonstrated in October where there was an increase of 0.4 per 100,000 in the rate of suicide (26). One difficulty in applying data from global platforms is the inherent differences in the management of the pandemic by each nation. Length/extent of lockdown restrictions, prevalence and mortality of COVID and economic factors will all contribute strongly to the mental resilience of citizens (23, 27). Therefore, it is difficult to generalise findings from one country to the global stage. When viewed collectively the literature remains inconsistent on the effect lockdown may have on suicidal behaviours within the UK, thus further research from multiple national centres to explore this question is justified.

Several other studies have utilised survey methods to investigate suicidal ideation and mental health more generally and have established more consistent trends. A UK study conducted in April 2020, which surveyed 17,452 individuals, showed an 18.9% increase in reports of psychological distress compared with the previous years (7). A survey from Spain found that COVID had a severe psychological impact on 30.4% of participants (8). These findings are echoed at a global level with articles from other countries such as Italy, China and Korea displaying similar worrying trends (9–11). These studies bolster the notion that subjective mental health has been negatively affected by the pandemic. What remains unclear is whether lockdown is the paramount contributing factor of these observations or if they translate into the increased manifestation of extreme behaviours such as DSH and suicide.

Perhaps the most concerning statistic discerned from our data is the rate of growth of presentations from March to May 2020 (111% increase in the raw number of admissions and a 128% increase in proportion of admissions). Conferring evidence to suggest that, as the length of lockdown increases, the burden upon mental health also sharply increases. This is particularly relevant now, during the formative stages of the new lockdown and acts as a warning of the potential psychological toll which may materialise in the coming months. It is therefore imperative that measures to combat this possible scenario are employed. One study from Italy postulated the role of increased access to teletherapy as a mechanism to empower individuals to alter their outlook on stressful situations thus partially alleviating their sense of anxiety (28). Indeed, some NHS trusts have already begun to champion this style of consultation and by raising awareness and increasing the availability of this service may help to remedy the effects of lockdown (29). Moreover, a study from Finland demonstrated that coping mechanisms which emanated from close personal relationships proved to be the most significant strategy to maintain psychological well-being during the COVID crisis (30). Therefore, by increasing access and education relating to virtual communication platforms, which allow family units to stay connected, may prove beneficial.

From a demographic perspective this study highlighted a statistically significant increase in the ratio of DSH cases which were male in 2020. This finding is contrary to not only historical data about DSH prior to the pandemic but also from studies conducted during COVID times. It is widely accepted that females are at an increased risk of DSH. A study from The Lancet referenced a 2.9% difference in the prevalence of DSH between sexes in 2014 (*p* = 0.0002) (31). This trend is further evidenced by the Adult Psychiatry Morbidity Survey conducted by NHS digital which found that 10% of women reported a severe common mental disorder compared with only 6% of men (32). This gender discrepancy has been further widened by the events stemming from COVID 19. A UK longitudinal study showed that the deterioration in psychological distress score was 6.9 percentage points higher in women than men in 2020 (33). Indeed, a UCL study expressly reports an increased tendency to self-harm amongst the female population during the initial lockdown period (34). This paper finds no overt reason why males in Manchester have demonstrated a higher risk of DSH behaviours compared to other populations. From March-April 2020, Manchester was subject to the same restrictions as other parts of the country and Government reports indicate both men and women were equally affected by the economic burden of COVID (35). Whilst this may represent a spurious occurrence, the significance emanating from the *p*-value warrants further investigation into possible factors which may be influencing this result. Although the average age did show a statistically significant increase in 2020, each cohort remained within the same general age bracket and therefore this finding was not deemed to be of clinical significance.

The number of observed overdoses involving paracetamol significantly decreased from 45.55%/48.46% of DSH admissions in 2018/2019, respectively to 30.51% in 2020. A possible underlying explanation for this finding stems from the accessibility of paracetamol in the second quarter of 2020. The UK Government's decision to enforce a lockdown in March 2020 sparked widespread fear within the general population. This fear was translated into a fierce survival instinct leading to the stockpiling of many products by individuals within society. Paracetamol was one such commodity and this resulted in sporadic shortages of the drug across many areas of the UK (36). Furthermore, the ability to gain access to paracetamol was further hampered by the closure of many high street shops consequentially coupled with the long queues originating from essential shops which remained open (37). Both factors listed above hindered the access to paracetamol and may have tempered the impulsivity associated with many cases of substance overdose.

There are some important limitations to this study. It is difficult to assess whether the increasing proportion of deliberate self-harm was due to imposed lockdown measures or from the direct effects of the virus. Many people have suffered unexpected bereavement or have themselves become deconditioned as a result of COVID infection. These factors are also likely to influence the mental resilience of the population and may contribute towards the observed increase outlined in this paper. Additionally, this may have perpetuated the observed trend in the rate of growth of admissions beyond the relaxation of lockdown in 2020 and represents a vital area of study for other papers. The data was gathered from one NHS trust in Manchester, a large metropolitan city known to struggle with higher-than-average rates of COVID 19 infection. It is therefore difficult to generalise our findings to the entire UK population. Further studies examining similar data from other regions would be useful in determining the scale of the problem identified in this study. Finally, due to the retrospective nature of this study, there is potential for bias, especially around case selection. Some may have been missed due to error in coding which may affect results.

## Conclusion

While it is undoubtably true that, from a public health perspective, control of the virus must be given paramount concern, it cannot be said that resources should not be devoted to mitigating the negative ramifications of these strategies. Our study has demonstrated a relative stability in the number of DSH admissions across the 3 years, despite a radical decline in all cause admissions in 2020. Moreover, this paper has found an accelerating trend of DSH admissions with increasing time under lockdown. This confers an unmet need for psychological support in the general population during these unprecedented circumstances and highlighted the need for prompt action to curtail the psychological harm which may ensue from future. Possible interventions which will help achieve this goal centre around increasing awareness and education around technologies which help to provide access to therapies and maintain vital support networks.

## Ethics Statement

This paper represents a service level audit involving usual care. In view of this review and approval by a research ethics committee was not required according to institutional or national guidelines. This was validated by an online tool provided by NHS Health Research Authority (38).

## Author Contributions

CS: lead author, data collection, study design, and article write up. AH and AW: contribution to writing and interpretation of data. DR: statistical analysis. OM and JB: data collection. All authors contributed to the article and approved the submitted version.

## Conflict of Interest

The authors declare that the research was conducted in the absence of any commercial or financial relationships that could be construed as a potential conflict of interest.
